# The Elastic Contact and Stability Analysis of an Inertial Micro-Switch with a Spring Stationary Electrode

**DOI:** 10.3390/s18124238

**Published:** 2018-12-03

**Authors:** Wenguo Chen, Huiying Wang, Dejian Kong, Shulei Sun

**Affiliations:** 1 The School of Information Engineering, Qujing Normal University, Qujing, Yunnan 655000, China; kingwhy2008@163.com (H.W.); xwkdj@126.com (D.K.); 2 The School of Mechanical Engineering, Guizhou Institute of Technology, Guiyang, Guizhou 550003, China; bobsunstuy@gmail.com

**Keywords:** MEMS, inertial micro-switch, surface micromachining, contact time, spring stationary electrode

## Abstract

A mechanical trigger inertial micro-switch with spring stationary electrode is proposed and fabricated by surface micromachining. The elastic contact process and stability performance are evaluated through experimental tests performed using a drop hammer. The test results show that the contact time is about 110 μs and 100 μs when the threshold acceleration is 480 g and the overload acceleration is 602 g, respectively. The vibration process of the electrodes is explained through an established physical mode. The elastic contact process is analyzed and discussed by Finite Element Analysis (FEA) simulations, which indicated that the contact time is about 65 μs when the threshold acceleration is 600 g. At the same time, this result also proved that the contact time could be extended effectively by the designed spring stationary electrode. The overload acceleration (800 g) has been applied to the Finite-Element model in ANSYS, the contact process indicated that the proof mass contacted with stationary electrode three times, and there was no bounce phenomenon during contact process, which fully proved that the stable contact process can be realized at high acceleration owing to the designed elastic stationary electrode.

## 1. Introduction

Inertial micro-switches are threshold accelerometers which can be used to realize the on-off state of a signal electrode when the moving electrode contacts the stationary electrode. As a passive device, the inertial micro-switch is widely used in wearable devices (low-g) and the Internet of Things [1,2,3], for example, a fall warning coat for the elderly (low-g), integrated installation systems in which is difficult to replace the batteries, the detection of high-speed collisions (high-g), and so on [4,5,6,7]. However, the disadvantages of short contact time and unstable contact restrict the applications of inertial switches. Firstly, the trigger signal of an inertia switch needs to be collected and processed by a pre-circuit before entering the drivers. When collecting signals, it is required that there should be a very large discrimination between the trigger signal and any noise signal. In other words, it is required that the frequency of the trigger signal be small (large pulse width) enough to distinguish the trigger signal from the noise signal, so as to avoid system misjudgments. Secondly, a stable and low-frequency trigger signal is conducive to signal processing by the pre-circuit. For example, when the comparator in the pre-circuit is used to capture the trigger signal and output a signal with equal pulse width, unstable signals can cause the signal to be ignored, and cause system misjudgments. Therefore, as the detection component in the integrated installation system, the inertial switch’s output stable long pulse signal can effectively improve the system reliability.

**Figure 1:**
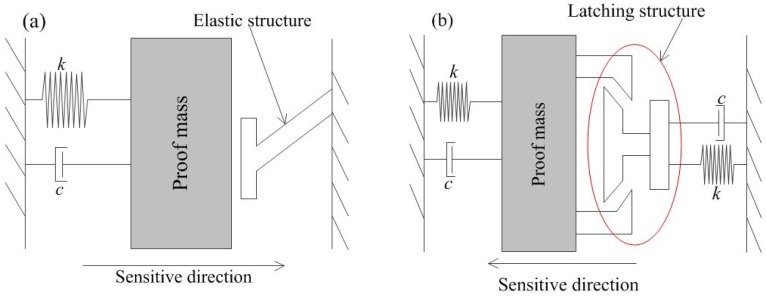
(**a**) The operating principle of flexible contact and (**b**) the principle of latching structure.

To achieve stable and low frequency trigger signals, two inertial switch structure design schemes are proposed. The first is a flexible contact scheme based on a non-silicon MEMS process from bottom to top [8,9,10], the other is a latching structure based on silicon etching [11,12]. The operating principles of above two designs are shown in Figure 1. The former design requires high stability of the device during the contact process, and the research results indicate that the low frequency and stable trigger signal can be obtained when the threshold acceleration is less than 100 g. However, the contact process is influenced by many factors such as frequency of impact load, threshold acceleration, and so on. Especially as the threshold increases, the trigger signal of the inertial switch with flexible electrode also becomes unstable and the contact time is short. For the latching structure design scheme, a latching acceleration switch with a threshold of more than 4000 g was proposed by Guo et al. The movable electrode latched into stationary electrode when the acceleration exceeded the set threshold, and the trigger signal was a step wave. The main fault of latching model, is that it was a disposable product, unsuitable for repeated use.

In view of the above questions, a novel longitudinal driven model with an elastic stationary electrode has been proposed for when the threshold is about 600 g. This device first reported in Ref. [13]. Compared to previous structures, the novel parts of this design can be described as follows: first, the longitudinal drive design is more stable than the horizontal one, because the longitudinal mode’s vibration direction of the movable electrode suspended by the symmetrical spring is vertically upward [14]. Second, the suspended structure with spring was proposed as the fixed electrode to improve the contact performance of the electrodes. Because the elastic deformation of stationary electrode causes the increase of adhesive displacement at the moment of electrode conduction, the contact time is extended. Third, in this structural design, the line and point contact between movable electrode and fixed electrode is achieved. Compared with the contact mode between the surface and the line, this novel design is more stable. In addition, the vibration transmission process of elastic contact and the signal output stability are addressed. The contact process is considered as a transmission of vibration, the vibration being transmitted from the movable electrode to stationary electrode when the applied acceleration amplitude exceeds its threshold. The theoretical analysis and test results indicate that the trigger signal of proposed inertial switch with a flexible stationary electrode is low frequency and stable, providing a reference for the design and manufacture of similar devices.

## 2. Structure Design

The inertial switch, also known as the threshold accelerometer, is a passive device that integrates sensing and execution. The main structure of the inertial switch consists of two parts: one is a movable electrode made up of mass blocks, and the other is a stationary electrode. The working principle can be expressed as follows: when the sensor is impacted by a vibration acceleration beyond the set threshold in the sensitive direction, the movable electrode is driven by inertial force to move towards the fixed electrode and make contact with it, so as to realize instantaneous conduction of the external circuit. When the half-sine wave acceleration is applied to the inertial switch, the threshold can be evaluated by Equation (1):

(1) $$a_{th}=x_{0}\frac{\left(k/m\right)-{\left(\pi /t_{0}\right)}^{2}}{\left(\sin\frac{2\pi }{\left(1+\sqrt{k/m}\left(t_{o}/\pi \right)\right)}-\frac{\left(\pi /t_{0}\right)}{\sqrt{k/m}}\sin\frac{2\pi \sqrt{k/m}}{\left(\pi /t_{0}\right)+\sqrt{k/m}}\right)}$$

where *x*_0_ is the gap between the electrodes, *t*_0_ is the pulse width of acceleration, *k* and *m* is the elastic coefficient and weight of the movable electrode.

**Figure 2:**
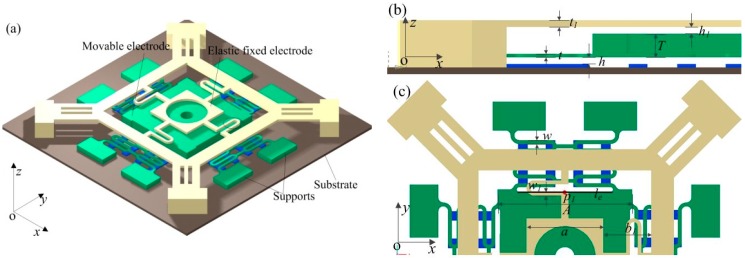
The structure diagrams of inertial micro-switch. (**a**) The 3D view of structure. (**b**) The side view of one-half structure. (**c**) The top view of one-half structure.

In order to prolong the contact time between electrodes and improve the contact stability, a new type of spring stationary electrode is presented in this paper. The whole structure of the device consists of three parts: many strips are symmetrically distributed on the substrate, which can weaken the effect of air damping in the *z* direction [15]. The proof mass is suspended by four groups of conjoined serpentine springs to operate as the movable electrode. The cross springs keep the stationary electrode suspended above the proof mass. In this model, the suspended stationary electrode is proposed based on the vibration transmission mechanism. The whole designed structure is shown in Figure 2a. A side view and top view of half the structure are shown in Figure 2b,c.

The gap between the proof mass and spring stationary electrode is set as 20 μm (*h* = 20 μm). In order to ensure that the displacement of spring stationary electrode tends to zero before a collision is detected, meanwhile, considering the process compatibility and the stiffness of the spring, the optimized stationary electrode parameters listed in Table 1 are selected. In other words, the displacement of the stationary electrode is entirely due to the impact of the movable electrode. A point on the stationary electrode (*P*_1_) is designed on the edge line (*l_e_*) of the proof mass as shown in Figure 2c. The movable electrode can move towards to the stationary electrode and touch the contact point when the acceleration amplitude reaches the designed threshold-level in the sensitive direction (*z* direction). Owing to the fact point *P*_1_ and the edge line (*l_e_*) are arranged collinearly, the point *P*_1_ will always keep in contact with *l_e_* during the contact process. This novel design provides the benefit of prolonging the contact time, while, enhancing the stability of the contact process. 

## 3. The Analysis and Simulation

### 3.1. Theoretical Analysis

**Figure 3:**
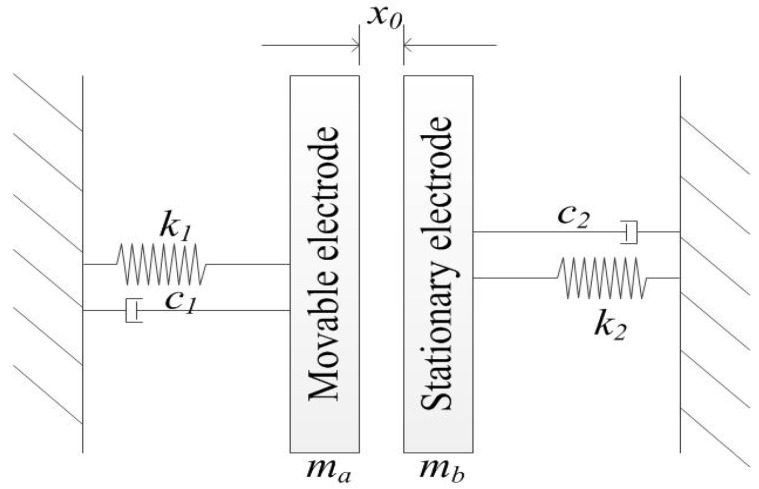
The physical model of the inertial micro-switch.

Both the movable and stationary electrode structures have suspended springs in the proposed model, which can be abstracted as a physical model consisting of two Single-Degree-of-Freedom (SDOF) systems as shown in Figure 3. The gap between movable and stationary electrodes is set as *x*_0_, the *k*_1_, *k*_2_, *c*_1_, *c*_2_ are the elastic constants and the damping coefficients of the movable and stationary electrodes, respectively. In this designed model, the movable electrode moves towards to stationary electrode when an acceleration load is applied to the inertial switch, and the stationary electrode vibrates when the movable electrode contacts it.

This vibration system requires that the vibration of the stationary electrode come entirely from the contact, so the sticking point is how to keep the stationary electrode in a silent state before the contact occurs. According to the parameter optimization, the question can solve satisfactorily, which is analyzed in the following paragraphs.

**Figure 4:**
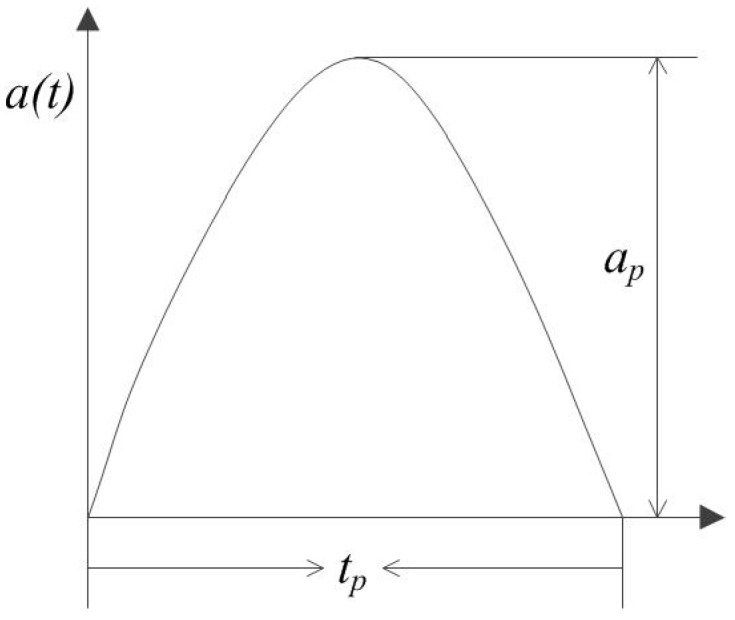
The half sine wave acceleration.

In practical use, the inertial switch is usually applied by the acceleration of a half sinusoidal waveform. Wherefore we analyze the dynamic response process of the designed inertial switch base on the half sine wave acceleration as shown in Figure 4.

The corresponding equation is:

(2) $$a(t)=\left\{ \begin{array}{ll} a_{p}\sin\omega t & t<t_{p}\\ 0 & t>t_{p} \end{array}\right.$$

where $a_{p}$ is amplitude, $t_{p}$ is pulse width, ω is the load frequency. Scale and align correctly.

When the acceleration load is applied to the inertial switch, vibration quickly transmits from the substrate to the spring electrode. The movable electrode is in a forced vibration state during the whole pulse. This time, the equilibrium equation of “mass-spring-damper” system can be expressed as follows [16]:

(3) $$m\frac{d^{2}x}{dt}+c\frac{dx}{dt}+kx=ma(t)=p(t)$$

where *m* is the weight of proof mass, *c* is damping coefficient, *k* is elasticity coefficient, *a*(*t*) is the acceleration over time, *p*(*t*) is the equivalent load.

**Figure 5:**
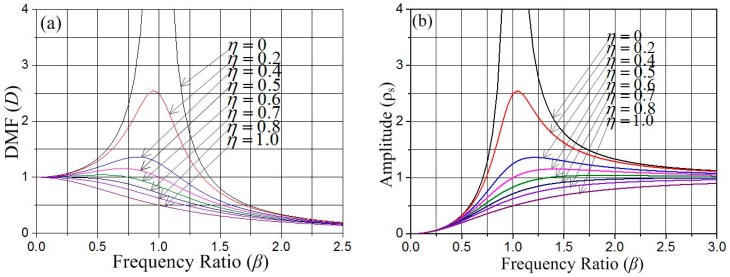
(**a**) The dependence of factor *D* on the frequency ratio *β*. (**b**) The dependence of amplitude *ρ_s_* on the frequency ratio *β*.

In general, the dynamic response process is affected by the elastic damping force. According to the damping ratio ($\eta =\frac{c}{2m\omega_{0}}$) of system, the standard form of the dynamic equilibrium equation of the vibration system can be written as:

(4) $$\frac{d^{2}x}{dt^{2}}+2\eta \omega_{0}\frac{dx}{dt}+\omega_{0}^{2}x=a(t)$$

where $\omega_{0}=\sqrt{\frac{k}{m}}$ is the intrinsic angular frequency of the vibration system. By analyzing Equation (4) with displacement conditions, the standard form of the dynamic equilibrium equation of vibration system is [17]:

(5) $$\rho =\frac{p_{0}}{k}{\left[{\left(1-\beta^{2}\right)}^{2}+{\left(2\eta \beta \right)}^{2}\right]}^{-1/2}$$

where *a*_0_ is static load, $\beta =\omega /\omega_{0}$ is the ratio of load frequency to natural frequency of the system, $x_{s}=\frac{p_{0}}{k}$ is defined as static displacement caused by *a*_0_. For a dynamic response process, the ratio between steady-state amplitude and static displacement can be expressed as:

(6) $$D=\frac{\rho }{x_{s}}={\left[{\left(1-\beta^{2}\right)}^{2}+{\left(2\eta \beta \right)}^{2}\right]}^{-1/2}$$

where *D* is defined as the dynamic magnification factor (DMF). The curves in Figure 5a illustrate the dependence of the DMF (*D*) on the frequency ratio (*β*), which indicates that the amplitude of the spring system can be magnified when the frequency ratio (*β*) does not exceed 0.75 and the damping ratio is less than 0.7.

Moreover, considering the dynamic response of inertial switch with acceleration load $a(t)=-\omega^{2}v_{a_{0}}\sin\omega t$, the equivalent load is $p(t)=m\omega^{2}v_{a_{0}}\sin\omega t$ (where $v_{a_{0}}$ is the initial velocity, which is a constant term). Thus, the equivalent equation for Equation (5) can be obtained as:

(7) $$\rho_{s}=D\cdot x_{s}=D\frac{m\cdot a(t)}{k}=\beta^{2}v_{a_{0}}D$$

From Equation (7), we have the response displacement on frequency ratio (*β*), and the dependence of $\rho_{s}$ on β is shown by the curves in Figure 5b.

As one can see in Figure 5, the damping ratio is a key factor that influences the dynamic behavior of the system. In this designed model, the air damping can be ignored due to the strip plate placed on the substrate, so the elastic damping force is the main contributor to the dynamic process. According to the results reported by Bao [18], *η* = 0.6 is chosen for this designed vibration system. Figure 5b indicates that the amplitude increases slightly due to the increase of the frequency ratio (*β*) when its value does not exceed ~1.25. The frequency response characteristics shown in Figure 5a are introduced to the designed vibration system, where the optimum process is that the movable electrode responds to the inertial shock during the forced vibration stage but the stationary electrode does not. By selecting optimal parameters, the natural vibration frequency of movable electrode is about 828 Hz, and the elastic constant *k*_1_ is about 75 Pa, as acquired from simulation results in ANSYS. The natural vibration frequency of the stationary electrode is about 1465 Hz, and the elastic constant *k*_2_ is about 2100 Pa. The frequency ratios of the load and the movable electrode, and the stationary electrode are about 0.6 and 0.03, respectively ($\beta_{mov}\approx 0.6$,$\beta_{fix}\approx 0.03$).

**Figure 6:**
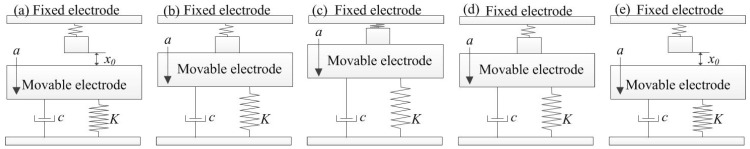
The vibrational transfer process and corresponding on-off state.

According to Figure 5b, one can reach the conclusion that the designed stationary electrode avoids the inertial impact during the forced vibration stage, as there is huge difference between $\beta_{mov}$ and $\beta_{fix}$ ($\beta_{mov}\gg \beta_{fix}$). The vibration of the stationary electrode comes absolutely from the contact with the movable electrode. The contact process of the electrodes is described in Figure 6.

The detailed process can be described as follows: (a) the inertial switch is considered as a vibratory conduction system, where the movable electrode moves to the stationary electrode when an acceleration is applied to the inertial switch; (b) the displacement of the proof mass reaches *x*_0_ while the acceleration reaches its threshold, meanwhile, the suspended stationary electrode remains insilence state before contact occurs; (c) the proof mass moves with the stationary electrode in the same direction, and the vibration transfers from the movable to the stationary electrode; (d) the proof mass and stationary electrode rebound to state (b) owing to the restorative force of the springs, and from state (b) to (d), the switch maintains the switch-on state to prolong the contact time; (e) finally, the proof mass returns to the equilibrium position after all the energy disappears due to free vibration. Therefore, one can obtain an effective contact-enhancing mechanism compared with conventional models. This mechanism indicates that the contact time is mainly derived from the elastic deformation: A greater amplitude will generate a larger elastic deformation, which will prolong the contact time. More importantly, there is no rebound during the contact process from (c) to (e) owing to the proposed novel stationary electrode structure.

### 3.2. Dynamic Contact Process Simulation 

**Figure 7:**
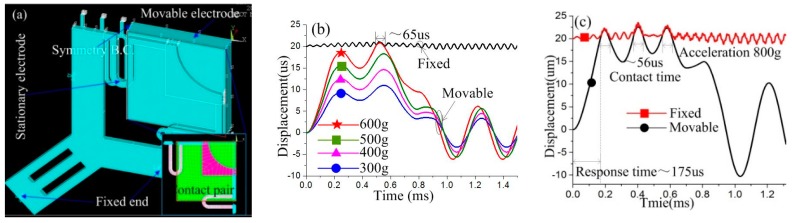
(**a**) The one quarter finite element model of designed inertial micro-switch. (**b**) The dynamic response curves of proof-mass and contact point under different acceleration loads. (**c**) The dynamic response curves of proof-mass and stationary electrode spring under an overload acceleration of 800 g.

To investigate the dynamic contact process, a finite element model (FEM) of the designed structure has been established with a 1:1 ratio using the ANSYS software. A quarter of the model was used to substitute for the whole one by applying symmetric boundary conditions to the symmetry plane. The one quarter model is shown in Figure 7a. All Degrees-of-Freedom (DOF) of the end section of springs and stationary electrode are constrained to zero. The top surface of the proof mass and the lower surface stationary electrode are defined as contact pairs. CONTA174 and TARGE170, SOLID 185 and SWEEP method are selected to mesh the model. Electroplated nickel (Ni) is selected as the structure and the properties of the material, including the Young’s modulus, and density are chosen as 165 GPa and 8.96 g·cm^−3^, respectively [19].

To evaluate the threshold acceleration of the designed model, accelerations from small to large (300, 400, 500 and 600 g) has been applied to the model in the sensitive direction. The dynamic vibration curves of the proof mass and stationary electrode spring are shown in Figure 7b. The simulation results indicate that the threshold acceleration of designed inertial micro-switch is about 600 g, the contact time is about 65 μs, and the response time is about 500 μs. The dynamic contact process indicates that the contact time can be prolonged owing to the elastic deformation of the stationary electrode spring.

**Figure 8:**
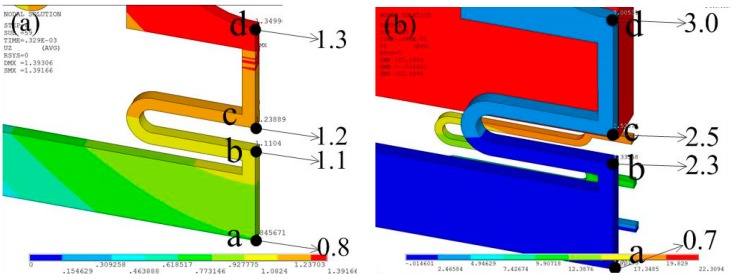
The maximum displacement of the stationary electrode when an overload acceleration of 800 g was applied. (**a**) The maximum displacement of the stationary electrode without contact. (**b**) The maximum displacement of the stationary electrode contacted by the proof mass.

**Figure 9:**
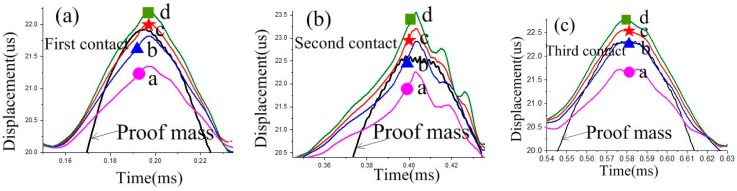
The response curves of points a, b, c d of the first, second and third contact. (**a**) First contact. (**b**) Second contact. (**c**) Third contact.

To evaluate the stability of the contact process, an overload acceleration (800 g) was applied to the designed inertial switch. The dynamic response process of the proof mass and stationary electrode is shown in Figure 7c. The dynamic process indicates that the proof mass contacts with the stationary electrode three times, and the maximum contact time is about 56 μs. The response time was about 175 μs. The dynamic contact process has been investigated by the maximum displacement of the stationary electrode spring, as shown in Figure 8. Figure 8a shows the maximum displacement of the stationary electrode without contact, which indicates that the location of the maximum displacement is point d, and it is 1.3 μm. The displacement gradually increases from point a to b. Figure 8b shows the maximum displacement of the stationary electrode owing to the contact of the proof mass, where the maximum displacement is point d, and it is 3.0 μm. Comparing Figure 8a with Figure 8b, the three following conclusions can be reached: (1) the displacement of the stationary electrode is mainly caused by the contact with the proof mass, the stationary electrode remains approximately silent if no contact occurs; (2) point c is the contact point during the dynamic process; (3) the deformation of the suspended spring is focused on the segment between point b and point c. The dynamic curves of points a, b, c, d of the first, second, and third contact are shown in Figure 9. The response curves of the three contacts continue to show that the vibration track of the proof mass between point b and c, the contact point is point c. The simulation results shown in Figure 8 and Figure 9 have demonstrated the contact process shown in Figure 6, which indicates that the contact process of designed inertial switch is stable.

## 4. Fabrication and Characterization

A prototype of the designed structure was fabricated by surface micromachining technology. Due to its excellent mechanical properties and material compatibility, nickel sulfate solution was chosen as the electroplating structural material. Based on the optimized individual technology, the structure of the device was electroplated orderly from bottom to top through a photoresist injection mold. The geometrical parameters of designed structure were measured by a stylus profiler in real time during all manufacturing processes. The condition parameters of the electroplating technique including solution temperature and pH, were chosen as 45 °C, 4.0, respectively. The deposition rate was controlled to be 0.38 μm/min. The main steps of the fabrication process can be described as follows: First, Cr/Cu was sputtered on the surface of glass as a seed layer; second, the photoresist was coated on metal films; third, the structure of the device was electroplated into the photoresist model; Fourth, the above steps were repeated until the entire device structure was prepared; finally, acetone was used to remove the photoresist, and an ammonia/peroxide solution was used to remove the metal film. The details are illustrated in Ref. [13].

**Figure 10:**
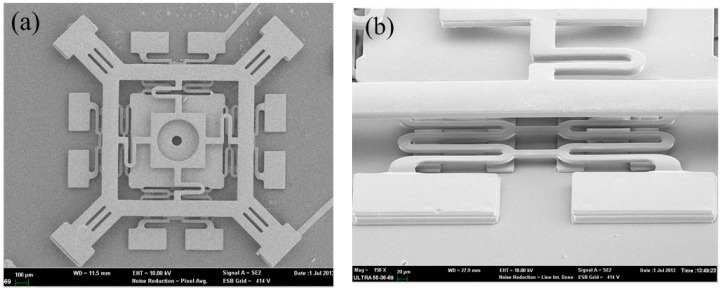
SEMs of the fabricated inertial micro-switch and a close-up. (**a**) The intact inertial micro-switch. (**b**) The suspended spring of the proof mass and stationary electrode cantilever.

Figure 10a shows the completed structure of the fabricated inertial micro-switch. Figure 10b shows the suspended spring of the proof mass and the stationary electrode cantilever. The SEMs of the prototype demonstrate that the designed inertial micro-switch can be fabricated successfully by surface micromachining.

**Figure 11:**
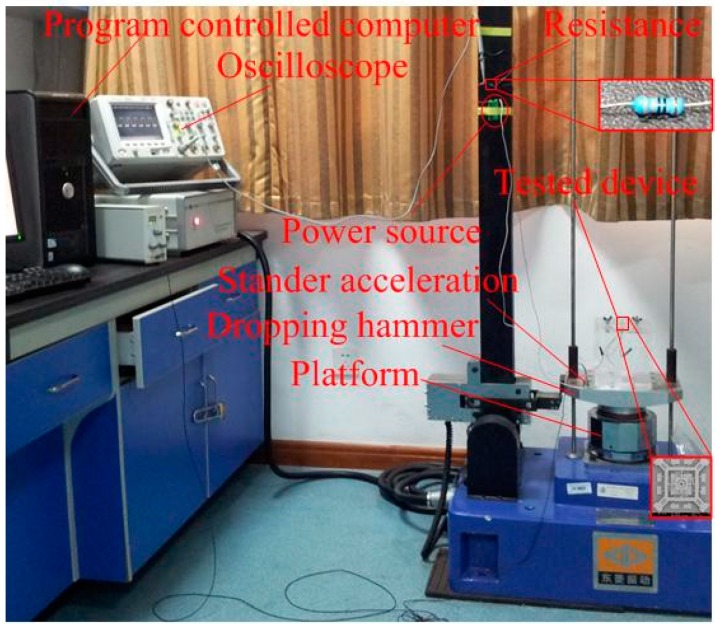
The dropping hammer system.

The fabricated prototype was tested by a dropping hammer system, as shown in Figure 11. In addition to the dropping hammer and computer, the main equipment of the testing system includes a standard accelerometer with a sensitivity of 9.8 mv∙g^−1^, a multichannel oscilloscope (6000 MSO6034A, Agilent, Penang, Malaysia), a DC power source and a 300 Ω current-limiting resistor. The prototype and accelerometer were stationary on the drop hammer. The sensitive direction of the fabricated prototype was installed perpendicularly to the ground. The pulse width of the applied acceleration was controlled by the stiffness of the pedestal. The acceleration applied to the testing prototype increased as the height of the dropping hammer rose from low to high, so the threshold of the designed inertial switch would be measured. The output acceleration curve is shown in the display and the corresponding signal is captured by the oscilloscope when the inertial switch is switched on.

**Figure 12:**
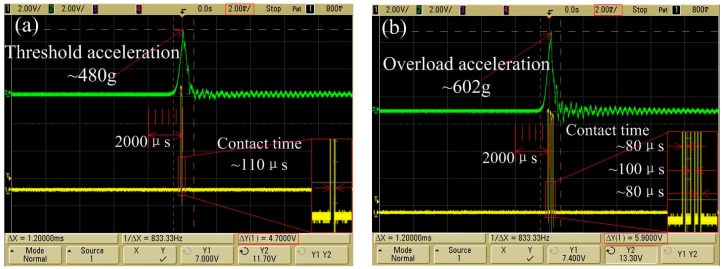
(**a**) The threshold acceleration curve and trigger signal. (**b**) The overload acceleration curve and trigger signal.

The threshold acceleration of the designed inertial micro-switch is 480 g with a pulse width of about 1 ms, as shown in Figure 12a. The contact time is about 110 μs, which indicates that the contact time of the designed inertial switch can be prolonged owing to the proposed stationary electrode. An overload acceleration had been applied to the prototype. The test result is shown in Figure 12b, which indicates that the the overload acceleration is about 602 g with a pulse width of about 1 ms. The contact time indicated that three contacts occurred, and the maximum contact time is about 100 μs. Through shocking experiments by applying an overload acceleration, the test results indicate that the dynamic contact process is consistent with the simulation result, which proved that the contact process of the proposed stationary electrode is stable. The dynamic process of the test results is in agreement with the simulation results, but the test results of the threshold acceleration and contact time disagree with the simulation results. The simulation and experimental results are compared in Table 2.

**Figure 13:**
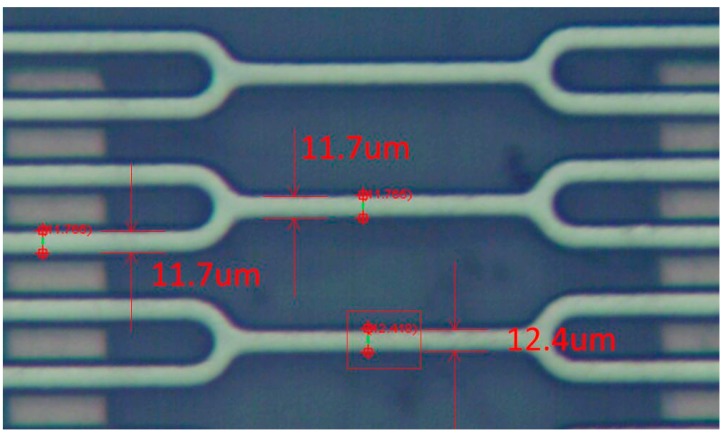
The line width of the suspended spring measured by microscope.

Preliminary analysis suggests that the test contact time is longer than in the simulation because of the effects of friction during the contact process. In addition, the reason why the test threshold is lower than the simulation is that the line width of the suspended spring is less than the design value. The measurement of the line width is shown in Figure 13. The result indicates that the actual value is about 12 μm while the design value is 15 μm, this is the main reason for the error compared with the simulation threshold.

## 5. Conclusions

A novel longitudinal driven inertial micro-switch with spring electrodes has been fabricated successfully based on surface micromachining technology. The dynamic contact process has been investigated by theoretical analysis, FE simulation and experimental methods. The theoretical analysis results indicate that the response process of electrodes can be controlled by optimizing the structure parameters. The simulation results indicate that the stability of the dynamic process can be improved effectively owing to the proposed stationary electrode, and the contact time is about 65 μs when the threshold acceleration is 600 g, and the maximum contact time is about 56 μs when the overload acceleration is 800 g. In an overload condition, three contacts occurred, and the analysis of the contact process indicated that the stability of the contact agreed with the principle. Finally, the fabricated prototype has been also tested by dropping hammer experiments, which indicated that the threshold acceleration of the designed inertial switch is about 480 g, and the contact time is about 110 μs. The maximum contact time is about 100 μs when the overload acceleration is 602 g. The test results verified the theoretical analysis and simulation results.

## Figures and Tables

**Table 1:** The main geometric parameters of the designed inertial micro-switch.

Components	Proof Mass			Gap		Movable Electrode			Stationary Electrode		
Geometric parameters	*A*	*a*	*T*	*h*	*h_1_*	*w*	*b*	*t*	*w_1_*	*b*_1_	*t*_1_
Values (µm)	700	400	60	20	20	15	163	10	30	250	20

**Table 2:** The simulation and experimental results of designed inertial micro-switch.

Performance		Acceleration (g)	Contact Time (μs)
Simulation	Threshold	600	65
	Overload	800	56
Test	Threshold	480	110
	Overload	602	100
